# Artificial Intelligence Perceptions and Technostress in Staff Radiologists: The Mediating Role of Artificial Intelligence Acceptance and the Moderating Role of Self-Efficacy

**DOI:** 10.3390/bs15091276

**Published:** 2025-09-18

**Authors:** Giovanni Di Stefano, Sergio Salerno, Domenica Matranga, Manuela Lodico, Dario Monzani, Valeria Seidita, Roberto Cannella, Laura Maniscalco, Silvana Miceli

**Affiliations:** 1 Department of Psychology, Educational Science and Human Movement, University of Palermo, 90133 Palermo, Italy; dario.monzani@unipa.it (D.M.); silvana.miceli56@unipa.it (S.M.); 2 Department of Health Promotion, Mother and Child Care, Internal Medicine and Medical Specialties, University of Palermo, 90133 Palermo, Italy; sergio.salerno@unipa.it (S.S.); domenica.matranga@unipa.it (D.M.); manuela.lodico@you.unipa.it (M.L.); laura.maniscalco04@unipa.it (L.M.); 3 Department of Engineering, University of Palermo, 90133 Palermo, Italy; valeria.seidita@unipa.it; 4 Department of Biomedicine, Neuroscience and Advanced Diagnostics, University of Palermo, 90133 Palermo, Italy; roberto.cannella@unipa.it

**Keywords:** artificial intelligence, technostress, AI acceptance, self-efficacy, radiology, technology adoption

## Abstract

This study examined how perceptions of artificial intelligence (AI) relate to technostress in healthcare professionals, testing whether AI acceptance mediates this relationship and whether self-efficacy moderates the formation of acceptance. Seventy-one participants completed measures of Perceptions of AI (Shinners), AI Acceptance (UTAUT), Self-Efficacy, and four technostress outcomes: Technostress Overall, Techno-Overload, Techno-Complexity/Insecurity, and Techno-Uncertainty. Conditional process analyses (PROCESS Model 7; 5000 bootstrap samples) were performed controlling for sex, age (years), and professional role (radiology residents, attending radiologists, PhD researchers). Perceptions of AI were directly and positively associated with Technostress Overall (b = 0.57, *p* = 0.003), Techno-Overload (b = 0.58, *p* = 0.008), and Techno-Complexity/Insecurity (b = 0.83, *p* < 0.001), but not with Techno-Uncertainty (b = −0.02, *p* = 0.930). AI Acceptance negatively predicted the same three outcomes (e.g., Technostress Overall b = −0.55, *p* = 0.004), and conditional indirect effects indicated significant negative mediation at low, mean, and high self-efficacy for these three outcomes. Self-efficacy moderated the Perceptions → Acceptance path (interaction b = −0.165, *p* = 0.028), with a stronger X→M effect at lower self-efficacy, but indices of moderated mediation were not significant for any outcome. The results suggest that perceptions of AI exert both demand-like direct effects and buffering indirect effects via acceptance; implementation should therefore foster acceptance, build competence, and address workload and organizational clarity.

## 1. Introduction

The integration of artificial intelligence (AI) technologies in healthcare represents a fundamental shift in how medical professionals deliver patient care, with radiologists being at the forefront of this technological transformation (Kelly et al., 2023). Healthcare institutions are increasingly leveraging AI-based systems to advance precision medicine approaches and improve efficiency and quality assurance (Fogel & Kvedar, 2018), with the rapid advancement of AI capabilities in image analysis and pattern recognition particularly transforming radiological practice (Kaur & Garg, 2023; Stoitsis et al., 2006; Zhang & Dahu, 2019). For radiologists, AI serves as a crucial tool in enhancing diagnostic accuracy, customizing patient care approaches, optimizing radiation exposure, and reducing interpretation errors in medical imaging (Derevianko et al., 2023; Pesapane et al., 2018). While extensive research has examined technology acceptance in healthcare through established frameworks such as the Technology Acceptance Model (TAM; Davis, 1989) and the Unified Theory of Acceptance and Use of Technology (UTAUT; Venkatesh et al., 2003, 2012), contemporary studies reveal that AI adoption creates unique psychological and organizational challenges that traditional models may not fully capture. Contemporary research highlights how healthcare professionals’ adoption of AI and related technologies is shaped by their assessment of its practical utility and accessibility (Kelly et al., 2023; Lambert et al., 2023; Schiavo et al., 2024), yet growing evidence suggests that individual competencies and stress-related responses play increasingly critical roles in determining successful adaptation outcomes (Wu et al., 2025a).

However, the current understanding of AI integration in healthcare faces a critical theoretical gap: while we know that technology acceptance models predict adoption behaviors, and separate research streams have identified technostress as a consequence of digital transformation, the mechanisms through which AI perceptions translate into stress outcomes through acceptance processes remain theoretically underdeveloped. Further investigation is needed to explore how healthcare providers’ experiences with AI implementation correlate with technology-induced stress (Malik et al., 2022; Yamamoto, 2019), particularly since AI adoption as a stress factor has only been explored in a limited number of studies (Chang et al., 2024). Chen et al. (2024) recently demonstrated that AI-driven technologies create novel forms of technostress that operate through distinct psychological pathways, yet the field lacks comprehensive models explaining how these stress responses emerge from the interaction between perceptions and acceptance behaviors. This gap is particularly problematic in radiology, where AI systems directly augment diagnostic decision-making, potentially creating unprecedented challenges to professional identity and competence. The failure to understand these mediating mechanisms has significant practical implications: radiologists experiencing high technostress may be less likely to effectively adopt and utilize AI systems, ultimately compromising diagnostic accuracy, patient care quality, and professional wellbeing. Moreover, without understanding how self-efficacy beliefs moderate these relationships, healthcare organizations lack evidence-based strategies for supporting successful AI integration and mitigating technology-induced stress among their diagnostic staff.

The purpose of this study is to examine how radiologists’ perceptions of AI influence technostress through the mediating role of AI acceptance, and to identify how self-efficacy beliefs moderate these relationships. Drawing on UTAUT and extending it with insights from technostress research and self-efficacy theory, we propose and test a moderated mediation model that explains the conditional indirect effects of AI perceptions on work-related stress outcomes. Our findings contribute to theory by integrating previously disconnected research streams and provide practical insights for managing AI implementation in diagnostic medicine, ultimately supporting both technological effectiveness and professional wellbeing in radiology departments.

## 2. Theoretical Foundation

### 2.1. Technology Acceptance in Healthcare: From TAM to UTAUT

Healthcare technology adoption has been extensively studied through technology acceptance frameworks, with TAM (Davis, 1989) and UTAUT (Venkatesh et al., 2003, 2012) serving as foundational models for understanding how medical professionals evaluate and adopt new technologies. TAM posits that perceived usefulness and perceived ease of use are primary determinants of technology acceptance, while UTAUT extends this framework by incorporating performance expectancy, effort expectancy, social influence, and facilitating conditions as key predictors of behavioral intention and usage behavior. In healthcare contexts, these models have demonstrated robust predictive power for various technologies, with multiple factors shaping technology integration including perceived ease of use, expected performance benefits, social context, and supporting infrastructure (Ammenwerth, 2019; Jain et al., 2022; Rouidi et al., 2022).

However, artificial intelligence presents unique characteristics that may challenge traditional acceptance models. Unlike conventional healthcare technologies that primarily automate administrative tasks or facilitate information access, AI systems in radiology directly augment clinical decision-making processes, potentially altering the fundamental nature of diagnostic work. Wu et al. (2025b) demonstrated that digital transformation in healthcare organizations requires consideration of both job demands and job resources, suggesting that AI integration may simultaneously create new stressors and opportunities for professional development. This dual nature of AI technology necessitates extending traditional acceptance models to account for both positive and negative psychological outcomes, as the same technological implementation may function as both a resource that enhances capabilities and a demand that increases cognitive and emotional burden.

### 2.2. Technostress in the Digital Healthcare Environment

Technostress, originally defined as negative psychological responses to information and communication technology use, represents a psychological state of discomfort and anxiety resulting from the use of technologies, involving both the stress of adapting to new technologies and the ongoing strain from continuous technological demands (Ayyagari et al., 2011; Tarafdar et al., 2007). Unlike general occupational stress, technostress specifically results from the demands, pressures, and constraints associated with technology use in work settings. In healthcare, technostress manifests through multiple dimensions including technology overload, technology invasion, technology complexity, technology insecurity, and technology uncertainty.

Recent research has identified AI-driven technostress as a distinct phenomenon requiring specialized theoretical attention. Chang et al. (2024) demonstrated that AI-induced technostress operates through unique mechanisms involving both challenge and hindrance stressors, with employees’ affective reactions and technical self-efficacy playing crucial moderating roles. Chen et al. (2024) further revealed that the relationship between AI perceptions and stress outcomes involves complex mediating processes that traditional technostress models have not adequately captured, particularly in high-stakes professional environments where decision-making accuracy is critical. This finding suggests that AI implementation in healthcare may generate novel forms of stress that require more sophisticated theoretical understanding than conventional technology-related stressors.

### 2.3. Self-Efficacy as a Boundary Condition

Individual confidence in one’s capabilities within particular professional contexts reflects domain-specific self-efficacy beliefs (Bandura, 1982; Wood & Bandura, 1989). Self-efficacy theory provides a crucial lens for understanding individual differences in responses to AI integration. Defined as individuals’ beliefs in their capabilities to execute behaviors necessary to produce specific performance outcomes, self-efficacy has been consistently identified as a key moderator of technology-related attitudes and behaviors. In the context of AI adoption, self-efficacy beliefs specifically relate to confidence in one’s ability to effectively utilize AI systems to accomplish work tasks and maintain professional competence. Wu et al. (2025a) demonstrated that individual technological competencies significantly influence how employees respond to digital transformation initiatives, with higher self-efficacy beliefs associated with more positive attitudes toward AI integration and reduced stress responses during technological transitions. The theoretical rationale for self-efficacy’s moderating role rests on its influence on cognitive appraisal processes: individuals with high AI-related self-efficacy are more likely to view AI integration as a manageable challenge rather than an overwhelming threat, leading to more positive acceptance attitudes and reduced technostress responses; conversely, employees who lack confidence in their self-efficacy may struggle to adjust to new AI settings and utilize AI tools proficiently (Chang et al., 2024; Kim & Lee, 2021).

### 2.4. Integrative Model and Research Framework

**Figure 1:**
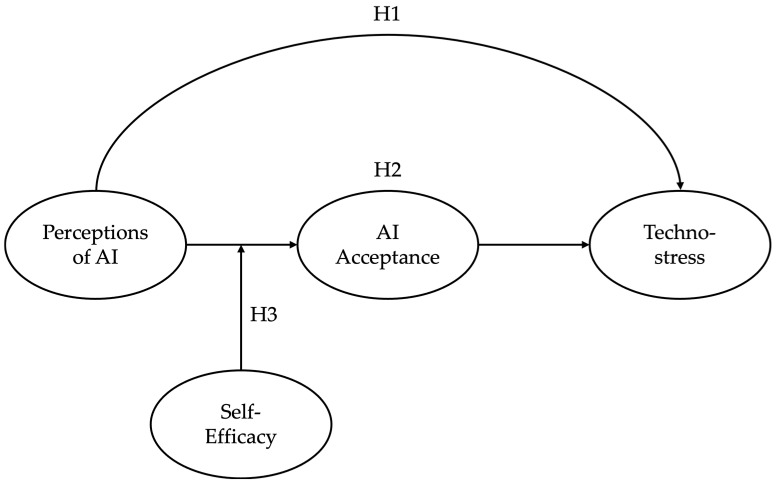
Proposed moderated mediation model.

Building on these theoretical foundations, we propose an integrative model that positions AI acceptance as a mediating mechanism between AI perceptions and technostress, with self-efficacy serving as a key boundary condition (see Figure 1). This model addresses the theoretical gap by explaining how positive perceptions of AI’s professional impact and one’s preparedness for AI use translate into reduced stress outcomes through enhanced acceptance behaviors. The moderating role of self-efficacy provides theoretical specification of when and for whom these relationships are strongest, addressing calls for more nuanced understanding of individual differences in AI adoption processes.

This theoretical integration contributes to the healthcare technology literature by demonstrating how established acceptance models can be extended to account for stress outcomes and individual competency factors. For clinical practice, the model provides a framework for understanding why some radiologists successfully adapt to AI integration while others experience significant stress and resistance, informing targeted intervention strategies for supporting successful AI implementation in diagnostic medicine.

## 3. Hypothesis Development

### 3.1. AI Perceptions and Technostress: A Job Demands-Resources Perspective

Understanding why positive perceptions of AI may reduce technostress requires a theoretical framework that can explain how technology functions as both a potential stressor and a resource. The Job Demands-Resources (JD-R) theory provides a compelling lens for this analysis, proposing that work factors can be conceptualized as either job demands (aspects that require sustained effort and lead to strain) or job resources (aspects that help achieve goals, reduce demands, and stimulate growth) (Bakker et al., 2023). According to JD-R theory, factors perceived as job resources result in work engagement and reduced stress, whereas factors perceived as job demands result in burnout and technostress.

In the context of AI integration in radiology, positive perceptions of AI’s professional impact and one’s preparedness for AI implementation are likely to result in viewing AI systems as job resources rather than job demands. When radiologists perceive AI as enhancing their diagnostic capabilities, improving accuracy, and supporting their professional development, they are more likely to experience the technology as a resource that augments their competence rather than a demand that threatens their autonomy (Benlian, 2020; Shinners et al., 2022). Professional resistance to AI adoption typically stems from concerns about its professional impact, perceived technical inadequacies, and insufficient organizational support, suggesting that positive perceptions serve as protective factors against technology-related stress.

Contemporary research supports this resource-oriented view of AI in healthcare. Studies indicate that when healthcare professionals perceive AI as beneficial to their clinical practice and feel adequately prepared for its implementation, they experience reduced technology-related anxiety and increased confidence in their ability to maintain professional competence (Lambert et al., 2023; Shinners et al., 2020). Conversely, negative perceptions of AI’s impact on professional autonomy and concerns about technical readiness have been associated with increased resistance and stress responses (Huisman et al., 2021; Waymel et al., 2019).

**H1.**  *Positive AI perceptions (perceived professional impact and implementation preparedness) are negatively related to technostress among radiologists.*

### 3.2. The Mediating Role of AI Acceptance

While direct relationships between perceptions and stress are important, understanding the mechanisms through which these effects occur provides deeper theoretical and practical insights. Technology acceptance literature, grounded in TAM (Davis, 1989) and UTAUT (Venkatesh et al., 2003, 2012), demonstrates that user acceptance plays a fundamental role in the effective integration of technological systems, including AI applications in professional settings. UTAUT identifies key determinants of technology adoption, including performance expectancy, effort expectancy, social influence, and facilitating conditions, with users’ confidence in a system’s effectiveness particularly shaping their adoption intentions.

The mediating role of AI acceptance in the relationship between AI perceptions and technostress can be understood through the lens of cognitive appraisal theory and technology acceptance models. When radiologists hold positive perceptions about AI’s professional benefits and their own preparedness, these perceptions enhance their behavioral intentions to accept and use AI systems (Davis, 1989; Venkatesh et al., 2003). Enhanced AI acceptance, in turn, reduces technostress by providing users with greater control over their technological environment and increased confidence in their ability to effectively utilize AI tools.

Healthcare professionals’ responses to AI technologies reveal this nuanced interaction between acceptance and stress factors (Califf et al., 2020). The introduction of AI systems can generate unique technological challenges and uncertainties (Issa et al., 2024), and healthcare professionals may experience technostress when using AI and other digital tools (Bail et al., 2023). However, factors influencing AI acceptance include perceived effects on error occurrence, alert sensitivity, and resource timeliness, while fear of loss of autonomy and integration difficulties hinder acceptance (Lambert et al., 2023). Insufficient technology acceptance can impair AI utilization, potentially undermining organizational innovation capacity and heightening staff vulnerability to technology-related stress (Ayyagari et al., 2011; Malik et al., 2022).

The logical progression from perceptions to acceptance to reduced stress aligns with established theoretical models. When healthcare professionals perceive feelings of incapacity and insecurity regarding AI, this negatively impacts technology acceptance and, in turn, leads to increased technostress. Conversely, positive perceptions enhance acceptance behaviors, which serve as a buffer against technology-induced stress by providing users with greater mastery and control over their technological environment.

**H2.**  *AI acceptance mediates the relationship between positive AI perceptions and reduced technostress, such that positive AI perceptions lead to increased AI acceptance, which in turn leads to reduced technostress.*

### 3.3. The Moderating Role of Self-Efficacy

Individual differences in responses to AI integration can be understood through self-efficacy theory, which provides crucial insights into when and for whom the relationships between AI perceptions, acceptance, and technostress are strongest. Self-efficacy, defined as individuals’ beliefs in their capabilities to execute behaviors necessary to produce specific performance outcomes (Bandura, 1982), represents a key individual difference variable that can moderate technology-related attitudes and behaviors.

The theoretical rationale for self-efficacy’s moderating role draws from Social Cognitive Theory’s (SCT) triadic reciprocal model, which argues that personal factors (such as self-efficacy perceptions) and external events (such as AI integration) interact to influence behaviors (such as AI acceptance) (Wood & Bandura, 1989). This interaction suggests that the relationship between AI perceptions and acceptance behaviors is contingent upon individuals’ confidence in their technological capabilities. Furthermore, UTAUT specifically positions self-efficacy not as having direct effects on behavioral intentions, but rather as a moderator that influences the strength of other relationships within the acceptance model (Venkatesh et al., 2003).

In the context of AI adoption in radiology, self-efficacy in AI use reflects radiologists’ confidence in their perceived proficiency in utilizing AI systems effectively. Studies indicate that professionals who possess strong self-efficacy regarding AI demonstrate enhanced ability to adapt to advancing technological systems, with higher trust in mastering new technologies and accomplishing work duties (Chang et al., 2024; Kim & Lee, 2021). Conversely, employees who lack confidence in their self-efficacy may struggle to adjust to new AI settings and utilize AI tools proficiently.

The moderating mechanism operates through cognitive appraisal processes: radiologists with high AI-related self-efficacy are more likely to view positive AI perceptions as actionable opportunities for professional enhancement, leading to stronger acceptance behaviors and greater stress reduction. In contrast, radiologists with low self-efficacy may be less able to translate positive perceptions into acceptance behaviors, potentially limiting the stress-reducing benefits of positive AI perceptions. This moderation effect is particularly important in healthcare contexts where professional competence and confidence are critical for both individual wellbeing and patient care quality.

**H3.**  *Self-efficacy in AI use moderates the relationship between AI perceptions and AI acceptance, such that the positive relationship between AI perceptions and AI acceptance is stronger for radiologists with higher self-efficacy compared to those with lower self-efficacy.*

All the hypotheses outlined earlier are summarized in the model presented in Figure 1.

## 4. Materials and Methods

### 4.1. Participants and Procedure

The study included 71 participants from the residency program in Radiology and Diagnostic Imaging at Palermo University, Italy. The participants consisted mainly of MD radiology residents (73.6%), with 22.2% being attending radiologists, and the remaining 4.2% being radiologists involved in PhD program. All participants were trained to use specific AI-driven diagnostic software in their clinical practice. In terms of gender distribution, 59.7% were male, 38.9% were female, and 1.4% did not to respond. The average age of the participants was 33.21 years (SD = 8.70 years).

A questionnaire was administered online from a secure web server from 1 September to 30 September 2024. Prior to accessing the questionnaire, participants were presented with an informed consent page. Only those who actively indicated their agreement by clicking “Yes” to the statement “I have read and understood the information and consent to participate in this research” were directed to the survey questions. This ensured that all data were collected exclusively from participants who had explicitly acknowledged understanding the study information and provided their informed consent to participate.

### 4.2. Measures

The questionnaire included scales evaluating perception toward AI, AI acceptance (derived from dimensions of UTAUT), self-efficacy in the use of AI, technostress dimensions, respectively. All items related to these scales adopted a five-point scale ranging from “1” (strongly disagree) to “5” (strongly agree).

Perceptions of AI. The attitudes and perception toward AI were assessed by Shinners Artificial Intelligence Perception (SHAIP) questionnaire (Shinners et al., 2022). This instrument, that consists of ten items, is designed to explore healthcare professionals’ perceptions of AI (sample item: “I believe that the use of AI in my specialty could improve the delivery of patient care”).

AI acceptance. The scale used to evaluate AI acceptance consists of 19 items from Venkatesh et al. (2012)’s UTAUT model scales, encompassing performance expectancy, effort expectancy, social influence, facilitating conditions, hedonic motivation, habit, and behavioral intention (sample item: “I would find the AI software useful for my work”).

Self-Efficacy in AI use. The self-efficacy beliefs in using AI have been evaluated by a four-item scale from Venkatesh et al. (2003) (sample item: “I could complete a job or task using the AI if there was no one around to tell me what to do”).

Technostress. Technostress was assessed with 18 items taken from the scale developed by Tarafdar et al. (2007), which includes the following dimensions: Techno-Complexity/Insecurity, Techno-Overload, and Techno-Uncertainty (sample item: “I am forced by this AI software to do more work than I can handle”).

### 4.3. Statistical Methods

Internal consistency was assessed using Cronbach’s alpha, with values above 0.70 considered acceptable, above 0.80 good, and above 0.90 excellent.

Following a two-step modeling approach (Anderson & Gerbing, 1988), confirmatory factor analysis (CFA) was used to evaluate the fit of the entire measurement model against competing alternative models using maximum likelihood estimation. First, for each one of the four constructs (i.e., perceptions of AI; AI acceptance; self-efficacy; technostress), the exploratory factor analysis (EFA) was conducted to identify the factor structure of the corresponding measurement items in our radiologists’ sample. The average score for each model was expressed as Mean (standard deviation). The Kaiser–Meyer–Olkin (KMO) was used to assess the sampling adequacy for each variable in the model and for the complete model. KMO values between 0.8 and 1 suggested the sampling was adequate. Scree plot and parallel analysis was conducted to detect the optimum number of factors for our sample. According to this method, the eigenvalues of the actual data are compared with the eigenvalues of randomly generated and resampled data. The optimal number of factors corresponds to the actual data factors whose eigenvalues are larger than the corresponding random/resampled data eigenvalues. In order to identify the obtained factors, we relied on standardized loadings above 0.50. Then, CFA was used to evaluate the fit of the entire measurement model against competing alternative models, using maximum likelihood estimation and multiple indices, recognizing that fit index interpretation should consider sample size and model complexity (Goretzko et al., 2024; Williams et al., 2004). Given our moderate sample size (*N* = 71) and complex measurement model, we evaluated fit using the following indices: Chi-square/df ratio (values ≤ 3 indicating reasonable fit), Root Mean Square Error of Approximation (RMSEA; values ≤ 0.08 indicating acceptable fit), Comparative Fit Index (CFI; values ≥ 0.90 indicating acceptable fit), Tucker–Lewis Index (TLI; values ≥ 0.90 indicating acceptable fit), and Standardized Root Mean Square Residual (SRMR; values ≤ 0.08 indicating acceptable fit). Additional comparative indices (AIC, BIC) were used to compare competing models, with Ilower values indicating better fit (Bentler, 1990). Specifically, a one-factor model (in which all items loaded onto a single factor) was compared with a series of models including an increasing number of factors, culminating in the model whose latent factor structure corresponded to the solution suggested by the EFA.

Hypothesis testing was conducted using moderated mediation analysis. Analyses were conducted using PROCESS macro for SPSS 26 and Process v4.2 (Hayes, 2022). PROCESS uses bootstrapping procedures with bias-corrected confidence intervals to test indirect effects, which is particularly appropriate given that indirect effects often violate normality assumptions (Hayes & Rockwood, 2020). The analysis estimated the indirect effect of AI perceptions on technostress through AI acceptance, the direct effect of AI perceptions on technostress (controlling for the mediator), and the total effect. The moderation effect was tested by examining the interaction between AI perceptions and self-efficacy in predicting AI acceptance. All reported conditional process analyses controlled for participants’ gender, age (years), and professional role (radiology residents, attending radiologists, and PhD researchers).

## 5. Results

The internal consistency of the questionnaire was excellent overall (alpha = 0.94), ranging across four domains between good values (alpha = 0.84 for Self-efficacy, alpha = 0.88 for Attitudes toward the AI) and excellent values (alpha = 0.96 for AI acceptance, alpha = 0.96 for Technostress).

The factorial structure of Perceptions of AI was a 2-factor structure with 58% of the overall inertia explained by the first factor and 42% explained by the second factor. The first factor described “Professional impact of AI”; the second factor explained “Preparedness to AI” as shown by standardized loading above 0.50. Only one item did not load on none of them. The factorial structure of AI acceptance was found as a 2-factor structure with 53% of the overall inertia explained by the first factor and 47% explained by the second factor. The first factor described “Motivation and intention of use”, since it correlated with items concerning the pleasure derived from using AI, the regular use of AI, and the future intention to continue doing so. The second factor explained “Perception of AI efficiency”, since it correlated with items regarding individual’s perception of the benefits of AI in improving work efficiency, the ease of AI utilization, and the proficiency in operating AI technology. Three items did not load well on any factor and hence were dropped from further analysis. The factorial structure of self-efficacy was confirmed as a 1-factor structure, with 58% of explained inertia. Finally, the factorial structure of technostress was a 3-factors structure with 44% of the overall inertia explained by the first factor, 34% explained by the second factor and 23% explained by the third factor. The first factor described technological “Complexity and Insecurity”, the second factor explained technological “*Overload*” and the third factor explained technological “Uncertainty”, being all loadings over 0.60. One item did not load well on any factor and hence was dropped from further analysis.

CFA was conducted to evaluate the fit of competing measurement models using maximum likelihood estimation. As shown in Table 1, we compared five different model specifications to identify the optimal factor structure for our data. The one-factor model, which assumed all items loaded on a single general factor, showed poor fit across all indices (χ^2^/df = 3.70, CFI = 0.254, TLI = 0.221, RMSEA = 0.19, SRMR = 0.219). Progressive improvements in model fit were observed as the number of factors increased (see Table 1).

The four-factor model, corresponding to our theoretical constructs (AI Perceptions, AI Acceptance, Self-Efficacy, and Technostress), demonstrated reasonable improvement (χ^2^/df = 2.83, CFI = 0.497, TLI = 0.472, RMSEA = 0.16, SRMR = 0.120). However, the best-fitting model was the four-factor second-order model (χ^2^/df = 2.17, CFI = 0.691, TLI = 0.671, RMSEA = 0.13, SRMR = 0.099), which specified hierarchical relationships with: (1) AI Perceptions as a second-order factor with Professional Impact and Preparedness as first-order dimensions, (2) AI Acceptance as a second-order factor with Motivation/Intention and Efficiency Perception dimensions, (3) Self-Efficacy as a unidimensional factor, and (4) Technostress as a second-order factor with Complexity/Insecurity, Overload, and Uncertainty dimensions.

Model comparison using information criteria confirmed the superiority of the four-factor second-order model (AIC = 7040, BIC = 7388) compared to alternative specifications, with lower values indicating better fit. While the absolute fit indices suggest room for improvement, the hierarchical model provided the best balance between theoretical interpretability and statistical fit given our sample size (*N* = 71) and model complexity (Goretzko et al., 2024).

The results of descriptive statistics are shown in Table 2.

For subsequent hypothesis testing, we maintained the multidimensional structure of technostress rather than using only the second-order factor. This decision was theoretically and empirically motivated: first, technostress research consistently identifies distinct mechanisms underlying different stress dimensions, with Complexity/Insecurity, Overload, and Uncertainty representing different pathways through which technology creates stress (Tarafdar et al., 2007); second, our EFA results demonstrated these dimensions had different explanatory power and factor loadings, suggesting they capture distinct variance in stress responses; third, examining dimension-specific relationships with AI perceptions and acceptance allows for more precise understanding of which aspects of technostress are most influenced by the hypothesized mechanisms, providing more nuanced insights for both theory and practice.

Hypotheses were tested using PROCESS macro Model 7 (Hayes, 2022) with 5000 bootstrap samples and bias-corrected 95% confidence intervals. Given the multidimensional nature of technostress, separate analyses were conducted for each dimension. Results are presented for Technostress Overall, Techno-Complexity/Insecurity, and Techno-Overload.

The first hypothesis predicted that Perceptions of AI would be directly associated with higher levels of technostress. Regression analyses showed significant positive direct effects of Perceptions of AI on Technostress Overall (b = 0.57, *p* = 0.003), Techno-Overload (b = 0.58, *p* = 0.008), and Techno-Complexity/Insecurity (b = 0.83, *p* < 0.001). In contrast, the direct effect on Techno-Uncertainty was not significant (b = −0.02, *p* = 0.930). These results partially support H1, indicating that perceptions of AI are positively associated with certain forms of technostress but not with uncertainty (see Table 3).

The second hypothesis proposed that AI Acceptance mediates the relationship between Perceptions of AI and technostress. Across models, AI Acceptance consistently emerged as a significant negative predictor of Technostress Overall (b = −0.55, *p* = 0.004), Techno-Overload (b = −0.56, *p* = 0.011), and Techno-Complexity/Insecurity (b = −0.83, *p* < 0.001). No significant effect was found for Techno-Uncertainty (b = 0.11, *p* = 0.639).

Conditional indirect effects further confirmed this mediation (see Table 4). Specifically, significant negative indirect effects of Perceptions of AI on technostress through AI Acceptance were found at low, mean, and high levels of self-efficacy (all CIs excluding zero) for Technostress Overall, Techno-Overload (except for high level of self-efficacy), and Techno-Complexity/Insecurity. For Techno-Uncertainty, the indirect effects were non-significant at all levels of self-efficacy (CIs including zero). Thus, H2 was supported for three out of four technostress outcomes.

The third hypothesis stated that Self-Efficacy moderates the relationship between Perceptions of AI and AI Acceptance. The interaction term was significant (b = −0.17, *p* = 0.028), indicating that the effect of Perceptions of AI on AI Acceptance decreased at higher levels of self-efficacy. Conditional effects analyses showed that the positive association between Perceptions of AI and AI Acceptance was strongest at low levels of self-efficacy (b = 0.67, *p* < 0.001) and weaker at high levels (b = 0.35, *p* = 0.022).

However, the index of moderated mediation was not significant for any technostress outcome (Table 5), as all confidence intervals included zero. This suggests that, although self-efficacy moderated the X → M path, it did not significantly moderate the overall indirect effect of Perceptions of AI on technostress via AI Acceptance. Thus, H3 was supported only in part.

## 6. Discussion

### 6.1. Theoretical Contributions

This study advances understanding of how radiologists’ perceptions of AI relate to different forms of technostress by integrating technology-acceptance and technostress studies within a moderated-mediation framework. Three main theoretical contributions emerge.

First, our findings highlight a complex dual pathway from Perceptions of AI to technostress. Perceptions of AI were positively related to several technostress dimensions (Technostress Overall, Techno-Overload, Techno-Complexity/Insecurity), but they were also associated with greater AI Acceptance, which in turn reduces technostress (negative indirect effects). This dual pattern—direct positive effects of perceptions on some stress dimensions together with negative indirect effects via acceptance—illustrates that perceptions of AI can act simultaneously as a perceived resource and a perceived demand, consistent with JD–R reasoning (Bakker et al., 2023). In other words, perceiving AI’s professional utility may increase willingness to accept the technology (a resource that buffers stress), while at the same time increasing awareness of task changes, workload, or skill demands that directly generate strain. This nuanced pattern complements prior calls to extend acceptance models to account for psychological outcomes beyond usage intentions (Califf et al., 2020; Lambert et al., 2023) and explains why studies that examine only total effects might miss competing mechanisms (Chen et al., 2024).

Second, by demonstrating AI Acceptance as a mediator between perceptions and technostress for three of the four technostress outcomes (Overall, Overload, Complexity/Insecurity), the results provide empirical support for the theoretical proposition that acceptance processes translate cognitive appraisals into affective/strain outcomes. Our results align with UTAUT-derived logic (Venkatesh et al., 2003, 2012) while extending it: acceptance not only predicts adoption behaviors but also functions as a protective mechanism that reduces technostress. This extends technology-acceptance theory in healthcare by linking acceptance to wellbeing-related outcomes rather than only to use or intention (Davis, 1989; Kelly et al., 2023).

Third, the role of Self-Efficacy clarified important boundary conditions, but not always as hypothesized. The interaction between Perceptions of AI and Self-Efficacy was significant in predicting AI Acceptance; however, contrary to the expectation that higher self-efficacy would strengthen the perceptions → acceptance link, the effect of Perceptions of AI on AI Acceptance was stronger at lower levels of self-efficacy (i.e., perceptions matter more for those with lower self-efficacy). Practically and theoretically, this suggests that for professionals who already feel confident with AI, acceptance is less contingent on their perceptions of AI’s professional impact—confidence may lead to acceptance regardless of perceived benefits—whereas for less confident professionals, positive perceptions are especially important to foster acceptance. This result nuances social-cognitive predictions (Bandura, 1982; Wood & Bandura, 1989) and recent findings about technical self-efficacy in AI contexts (Chang et al., 2024; Wu et al., 2025a): self-efficacy may reduce reliance on external evaluative cues (perceptions) to form acceptance judgments. Importantly, though, the index of moderated mediation was not significant for any technostress outcome. That is, Self-Efficacy moderated the first stage (X → M) but did not significantly change the overall mediated (X → M → Y) pathway in bootstrap tests—suggesting the moderation affects how acceptance is formed but does not reliably alter the downstream buffering role of acceptance in reducing technostress in this sample (Hayes, 2022).

Finally, the results reveal heterogeneity across technostress dimensions. Techno-Uncertainty did not show either direct or mediated associations with Perceptions of AI, suggesting that uncertainty may be governed by different antecedents (e.g., organizational unpredictability, AI explainability, or external regulatory contexts) than overload and complexity. This aligns with literature arguing that distinct technostress facets operate through separate psychological pathways and have different predictors (Issa et al., 2024; Tarafdar et al., 2007). The multidimensional perspective used here thus yields a more precise theoretical mapping of how AI-related attitudes cascade into stress.

### 6.2. Practical Implications

These findings indicate that organizations should target AI acceptance not only to drive uptake but also as a means to protect staff wellbeing: concrete, evidence-based demonstrations of clinical benefits and clear alignment with existing workflows help translate favorable perceptions into genuine acceptance, which in turn reduces overload and perceived complexity. Training and communication should be calibrated to users’ self-efficacy: less confident practitioners will benefit most from scaffolded, hands-on training, peer testimonials and mentoring, whereas more self-efficacious users require smooth integration, opportunities for customization, and roles as peer champions. Because overload and complexity/insecurity were the technostress dimensions most responsive here, practical efforts must combine usable, explainable interfaces (e.g., decision-support outputs with clear justifications) with workload-sensitive implementation strategies such as protected training time, temporary staffing adjustments, and participatory co-design of workflows. By contrast, techno-uncertainty appears to demand structural remedies—transparent governance, explicit clinical guidelines, and communicated timelines—rather than only individual-level persuasion. Finally, implementation should be iterative and diagnostic: monitor technostress subdimensions separately, use mixed methods to collect rapid feedback, and adapt training, design, and policy in response to which stress pathways emerge locally.

## 7. Limitations and Future Research

This study has several limitations that need to be mentioned. First, all parameters in our research were based on self-reports, which could lead to common method variance. Further investigation could alleviate the common method bias concern by incorporating responses from multiple sources, for example, utilizing supervisor appraisals rather than self-reported surveys to gauge workers’ AI adoption behavior. Furthermore, we should acknowledge the fluctuating and complex nature of employees’ emotions daily due to stress from technology; this limitation has the potential to hinder a full understanding of the effects of technostress on employees’ emotional wellbeing over time. Long-term daily surveys are required for future research to track the evolving impact of stress caused by AI use. Also, this research was from a single institution, a tertiary center in which AI is currently used in clinical practice. So, this cannot reflect the impact of AI in radiologists that need to face this technology for the first time. Last, this research has taken into consideration only the individual level; future investigations need to identify also contextual factors. Contextual variables (e.g., organizational culture, social support) may play a larger role in influencing AI acceptance than self-confidence alone. Future research could explore these factors further to understand their role in technology adoption within healthcare settings.

When interpreting the results of the CFA, it is important to consider the limited sample size available in this study, which may account for the marginally acceptable goodness-of-fit statistics. As highlighted by Hu and Bentler (1999), when the sample size is 250 or fewer, the joint use of absolute and relative fit indices may lead to an increased likelihood of model rejection, even when the model is reasonably specified.

## Figures and Tables

**Table 1:** Goodness-of-fit statistics and constructs specification for fitted CFA Models.

Measurement Models	Chi^2^ (df)	RMSEA	(90% CI)	AIC	BIC	CFI	TLI	SRMR
One-factor ^a^	3994 * (1081)	0.19	0.19–0.20	9476	9797	0.254	0.221	0.219
Two-factor ^b^	3289 * (1080)	0.17	0.16–0.18	8778	9101	0.433	0.408	0.146
Three-factor ^c^	3196 * (1078)	0.17	0.16–0.17	8685	9013	0.457	0.432	0.142
Four factor ^d^	3037 * (1074)	0.16	0.15–0.17	8537	8879	0.497	0.472	0.120
Four-factor 2nd order ^e^	2116 * (973)	0.13	0.12–0.14	7040	7388	0.691	0.671	0.099

Note: *N* = 71. ^a^ All items loaded on a single general factor. ^b^ (1) A combined factor including Perceptions of AI, AI Acceptance and Self-Efficacy items, and (2) Technostress items as a separate factor. ^c^ (1) Self-Efficacy as a separate factor, (2) Perceptions of AI and AI Acceptance items combined into a single factor, and (3) Technostress as a separate factor. ^d^ Items were assigned to four distinct first-order factors corresponding to the theoretical constructs: Perceptions of AI, AI Acceptance, Self-Efficacy and Technostress, with correlations estimated between all factors. ^e^ A hierarchical structure with four main factors: (1) Perceptions of AI as a second-order factor with two first-order subdimensions (Professional Impact to AI and Preparedness to AI), (2) AI Acceptance as a second-order factor with two first-order subdimensions (Motivation and intention of use and Perception of AI efficiency), (3) Self-Efficacy as a first-order factor, and (4) Technostress as a second-order factor with three first-order subdimensions (Techno-Overload, Techno-Complexity and Techno-Insecurity, and Techno-Uncertainty). * *p* < 0.001.

**Table 2:** Means, standard deviations and intercorrelations among variables.

	Variables	Mean	SD	1	2	3	4	5	6
1	Perceptions of AI	3.64	0.73						
2	AI Acceptance	3.68	0.72	0.666 *					
3	Self-Efficacy	3.32	0.97	0.426 *	0.414 *				
4	Technostress Overall	2.47	0.84	0.200	−0.112	0.133			
5	Techno-Overload	2.16	0.96	0.180	−0.100	0.051	0.868 *		
6	Techno-Complexity/Insecurity	2.35	1.00	0.224	−0.174	0.149	0.943 *	0.757 *	
7	Techno-Uncertainty	3.11	0.98	0.037	0.092	0.107	0.628 *	0.385 *	0.415 *

Note: *N* = 71. * *p* < 0.01.

**Table 3:** Regression results for the effects of Perceptions of AI and AI Acceptance on technostress.

Outcome	Predictor	b	SE	t	*p*	LLCI	ULCI
Technostress Overall							
	Perceptions of AI	0.57	0.18	3.13	0.003	0.21	0.93
	AI Acceptance	−0.55	0.18	−2.98	0.004	−0.91	−0.18
Techno-Overload							
	Perceptions of AI	0.58	0.21	2.73	0.008	0.16	1.00
	AI Acceptance	−0.56	0.21	−2.62	0.011	−0.98	−0.13
Techno-Complexity/Insecurity							
	Perceptions of AI	0.83	0.20	4.07	0.001	0.42	1.23
	AI Acceptance	−0.83	0.21	−4.04	0.001	−1.24	−0.42
Techno-Uncertainty							
	Perceptions of AI	−0.02	0.22	−0.09	0.930	−0.46	0.42
	AI Acceptance	0.11	0.22	0.47	0.639	−0.34	0.55

Note: *N* = 71. Models control for sex, age, and professional role. Covariate coefficients are omitted here for parsimony. b = unstandardized regression coefficient; SE = standard error; LLCI and ULCI = lower and upper bound of the 95% bootstrap confidence interval.

**Table 4:** Conditional indirect effects of Perceptions of AI on technostress outcomes through AI Acceptance, at low (−1 SD), mean, and high (+1 SD) levels of Self-Efficacy.

Outcome	Self-Efficacy	Indirect Effect	BootSE	BootLLCI	BootULCI
Technostress Overall	Low (−1 SD, 2.34)	−0.36	0.14	−0.65	−0.11
	Mean (3.32)	−0.28	0.12	−0.53	−0.09
	High (+1 SD, 4.29)	−0.19	0.13	−0.49	−0.00
Techno-Overload	Low (−1 SD, 2.34)	−0.37	0.15	−0.68	−0.08
	Mean (3.32)	−0.28	0.12	−0.55	−0.07
	High (+1 SD, 4.29)	−0.19	0.13	−0.50	0.01
Techno-Complexity/Insecurity	Low (−1 SD, 2.34)	−0.55	0.18	−0.90	−0.20
	Mean (3.32)	−0.42	0.15	−0.75	−0.18
	High (+1 SD, 4.29)	−0.29	0.17	−0.68	−0.01
Techno-Uncertainty	Low (−1 SD, 2.34)	0.07	0.16	−0.27	0.37
	Mean (3.32)	0.05	0.12	−0.22	0.28
	High (+1 SD, 4.29)	0.04	0.10	−0.19	0.21

Note: *N* = 71. Indirect effects are estimated from models that control for sex, age, and professional role; covariate coefficients are omitted from this table for parsimony. Bootstrapped CIs (5000 samples) are shown. Effects are significant when the 95% CI does not include zero.

**Table 5:** Index of moderated mediation for the conditional indirect effects of Perceptions of AI on technostress outcomes through AI Acceptance, moderated by Self-Efficacy.

Outcome	Index	BootSE	BootLLCI	BootULCI
Technostress Overall	0.09	0.07	−0.07	0.21
Techno-Overload	0.09	0.07	−0.07	0.22
Techno-Complexity/Insecurity	0.14	0.10	−0.10	0.30
Techno-Uncertainty	−0.02	0.05	−0.12	0.07

Note: *N* = 71. The index of moderated mediation is significant when the 95% bootstrap confidence interval does not include zero.

## Data Availability

The data presented in this study are available upon reasonable request from the corresponding author.

## Abbreviations

The following abbreviations are used in this manuscript:

AI	Artificial Intelligence
AIC	Akaike Information Criterion
BIC	Bayesian Information Criterion
CFA	Confirmatory Factor Analysis
CFI	Comparative Fit Index
EFA	Exploratory Factor Analysis
JD-R	Job Demands-Resources
KMO	Kaiser–Meyer–Olkin
RMSEA	Root Mean Square Error of Approximation
SRMR	Standardized Root Mean Square Residual
TAM	Technology Acceptance Model
TLI	Tucker–Lewis Index
UTAUT	Unified Theory of Acceptance and Use of Technology
